# Viral etiologies of lower respiratory tract infections in children < 5 years of age in Addis Ababa, Ethiopia: *a prospective case–control study*

**DOI:** 10.1186/s12985-023-02131-x

**Published:** 2023-07-23

**Authors:** Fiseha Wadilo, Adey Feleke, Meseret Gebre, Wude Mihret, Tamrayehu Seyoum, Kalkidan Melaku, Rawliegh Howe, Andargachew Mulu, Adane Mihret

**Affiliations:** 1 Armauer Hansen Research Institute, Ministry of Health, Addis Ababa, Ethiopia; 2 Department of Biomedical Sciences, College of Natural and Computational Sciences, Addis Ababa University, Addis Ababa, Ethiopia; 3 Department of Medical Laboratory Sciences, College of Health Sciences and Medicine, Wolaita Sodo University, Wolaita Sodo, Ethiopia; 4 Department of Microbiology, Immunology, and Parasitology, School of Health Sciences, Addis Ababa University, Addis Ababa, Ethiopia

**Keywords:** Lower respiratory tract infections, Respiratory viruses, Children, Ethiopia

## Abstract

**Background:**

Lower respiratory tract infections (LRTIs) are a major cause of morbidity and mortality in children worldwide and disproportionally affect Sub-Saharan Africa. Despite the heaviest burden of LRIs in Ethiopia, to date, no published studies have reported a comprehensive viral etiology of LRTIs among children in Ethiopia. The objective of this study was to determine and estimate the etiological contribution of respiratory viruses to LRTIs in < 5 years children in Ethiopia.

**Methods:**

A prospective case–control study was conducted from September 2019 to May 2022 in two major governmental hospitals, St. Paul Hospital Millennium Medical College and ALERT Hospital in Addis Ababa, Ethiopia. Nasopharyngeal/oropharyngeal samples and socio-demographic and clinical information were collected from children under 5 years. A one-step Multiplex real-time PCR (Allplex™ Respiratory Panel Assays 1–3) was done to detect respiratory viruses. STATA software version 17 was used for the data analysis. We computed the odds ratio (OR), the attributable fraction among exposed (AFE) and the population attributable fraction (PAF) to measure the association of the detected viruses with LRTIs.

**Results:**

Overall, 210 LRTIs cases and 210 non-LRTI controls were included in the study. The likelihood of detecting one or more viruses from NP/OP was higher among cases than controls (83.8% vs. 50.3%, *p* = 0.004). The multivariate logistic regression showed a significantly higher detection rate for RSV A (OR: 14.6, 95% CI 4.1–52.3), RSV B (OR: 8.1, 95% CI 2.3–29.1), influenza A virus (OR: 5.8, 95% CI 1.5–22.9), and PIV 1 (OR: 4.3, 95% CI 1.1–16.4), among cases when compared with controls. The overall AFE and PAF for RSV A were (93.2% and 17.3%), RSV B (87.7% and 10.4%) and Influenza A virus (82.8% and 6.3%), respectively. The mean CT values were significantly lower for only RSV B detected in the case groups as compared with the mean CT values of RSV B detected in the control group (*p* = 0.01).

**Conclusions:**

RSV, Influenza A and PIV 1 viruses were significantly associated with LRTIs in < 5 years children in Addis Ababa, Ethiopia. Therefore, we underscore the importance of developing prevention strategies for these viruses in Ethiopia and support the importance of developing and introducing an effective vaccine against these viruses.

**Supplementary Information:**

The online version contains supplementary material available at 10.1186/s12985-023-02131-x.

## Background

Lower respiratory tract infections (LRTIs) are a major cause of morbidity and mortality in children worldwide. In 2019, LRTIs contributed 13.9% of the 5.30 million deaths among children younger than 5 years, and were the primary cause of mortality among children aged 1–59 months [1]. Low socio-demographic index regions, like Sub-Saharan Africa, had the heaviest burden of LRTIs, which contributed to 50% of these deaths [2, 3].

After the introduction of the pneumococcal conjugate vaccine (PCV) and *Haemophilus influenzae* type b (Hib) vaccine, mortality due to bacterial LRTIs has decreased globally, and hence virus-associated LRTIs are likely to comprise an increasing proportion [4]; however, laboratory diagnosis of viral LRTIs remains challenging despite advances in diagnostic laboratory technology [5, 6]. Furthermore, distinguishing colonization from infection is an important factor in making the correct diagnosis of LRTIs [7]. To solve this diagnostic challenge, several studies use a case–control study design and compare the infection status of people with LRTIs (cases) to people without LRTIs (controls) [6, 8–13]. A number of studies have detected respiratory syncytial virus, influenza, and human metapneumovirus more commonly in cases than in controls [8, 10–13]. The case–control study design approach can help to solve the diagnostic issue at a population level, but it has been used only rarely in etiological studies [5].

Virus load in the nasopharyngeal could also help in distinguishing colonization from infection and could further predict disease severity. Several studies reported that LRTIs cases have a higher nasopharyngeal viral density (lower CT value) than asymptomatic children or children with moderate respiratory illnesses [14–21]. For instance, a multicenter study conducted in nine developing countries reported a higher mean viral load for adenovirus, Human bocavirus, Human metapneumovirus, parainfluenza virus 1, parainfluenza virus 3, rhinovirus, and respiratory syncytial virus in nasopharyngeal/oropharyngeal swabs from children with pneumonia than without pneumonia [16]. Similarly, a study conducted among children < 5 years old with severe respiratory illness in Kenya reported a lower mean CT value for RSV among cases (27.2) than asymptomatic controls (35.8, *p* = 0.008) [19].

A better understanding of the contribution of specific respiratory viruses to childhood LRTIs is needed to guide clinical management and preventive measures. Despite the heaviest burden of LRTIs in Sub-Saharan Africa, to date, we have found no published studies reporting comprehensive viral etiologies of LRTIs among children. Therefore, we conducted this prospective case–control study in Ethiopia to estimate the contribution of respiratory viruses to LRTIs among hospitalized children younger than 5 years.

## Methods

### Study area and population

A prospective case–control study was conducted from September 2019 to May 2022 in two major governmental hospitals (St. Paul Hospital Millennium Medical College and ALERT Comprehensive Specialized Hospital) in Addis Ababa, Ethiopia. Data collection was interrupted from February 2020 to July 2020, during the COVID-19 Pandemic.

Cases were under five year children with LRTIs (an acute respiratory illness with a history of fever or measured fever of ≥ 38 °C and cough, with onset within the past 10 days, requiring hospitalization”) [18]. Controls were also under five children admitted in the same hospital for diseases other than respiratory infections (children who did not meet the case definition for LRTIs). Cases and controls were excluded if they were above the age of 60 months. Children with LRTIs with an onset of more than 10 days were also excluded from the cases. Both cases and controls were enrolled throughout the study period using the marginal frequency of matching by age group and month of sample collection with cases.

### Data collection

Experienced pediatric nurses, in collaboration with pediatricians, identified eligible cases and controls, obtained informed consent from parents/guardians, collected sociodemographic and clinical information, and collected Naso/Oropharyngeal swab samples.

### Naso/Oropharyngeal swab collection

Naso/Oropharyngeal swabs were collected from all enrolled children. Nasopharyngeal specimens were collected by inserting flocked swabs (Copan) into the posterior nasopharynx and rotating 180° for 2–3 s [16]. Oropharyngeal specimens were then collected by MWE Σ Swabs (MWE) over both tonsillar pillars and the posterior oropharynx for several seconds. Following collection, swabs were placed together in the same 3-mL vial of universal transport media (SIGMA VCM™) [16]. After the NP/OP samples were collected from both cases and controls, samples were transported to the Armauer Hansen Research Institute (AHRI) and stored at − 80 °C until tested.

### Laboratory procedures

Nucleic acid from the Naso/Oropharyngeal samples was extracted manually with Ribospin_vRD Viral RNA/DNA Extraction kit (GeneAll, South Korea), using the manufacturer’s protocol. Briefly, 300 μl samples (swab-storage media) were transferred to a 1.5 ml microcentrifuge tube. Then 500 μl buffer was added to the tube and incubated for 10 min at room temperature to lyse the sample. Seven hundred μl buffer RB1 was further added to the lysate then the mixture was transferred to a mini-column. Finally, the pass-through of the mini-column was discarded and 30–50 μl of nuclease-free water was added to the center of the membrane in the mini-column. The purified nucleic acid was stored at − 80 °C. After extraction, the detection of respiratory viruses was done using Allplex™ Respiratory Panel 1–3 Assays (Table 1) (Seegene, South Korea). Amplification was performed using a CFX96 thermocycler (Biorad, Hercules CA, USA). PCR setup and results analysis were managed by CFX real-time PCR detection system (CFX ManagerTM Software-IVD v1.6). For each virus, a PCR Ct value ≤ 42 was used to define positivity.

**Table 1:** Allplex™ Respiratory Panel 1–3 Assays Analytes

Allplex™ Respiratory panel 1	Allplex™ Respiratory panel 2	Allplex™ Respiratory panel 3
Influenza A virus (Flu A)	Adenovirus (AdV)	Bocavirus 1/2/3/4 (HBoV)
Influenza A-H1 (Flu A-H1)	Enterovirus (HEV)	Coronavirus 229E (229E)
Influenza A-H1pdm09 (Flu A-H1pdm09)	Metapneumovirus (MPV)	Coronavirus NL63 (NL63)
Influenza A-H3 (Flu A-H3)	Parainfluenza virus 1 (PIV 1)	Coronavirus OC43 (OC43)
Influenza B virus (Flu B)	Parainfluenza virus 2 (PIV 2)	Human rhinovirus (HRV)
Respiratory syncytial virus A (RSV A)	Parainfluenza virus 3 (PIV 3)	Internal Control (IC)
Respiratory syncytial virus B (RSV B)	Parainfluenza virus 4 (PIV 4)	
Internal Control (IC)	Internal Control (IC)	

To detect severe acute respiratory syndrome coronavirus 2 (SARS-CoV2), we used Real-Time Fluorescent RT-PCR Kit, BGI Biotechnology (Wuhan) Co.Ltd, China. The cut-off value for a positive test was cycle threshold (Ct) value ≤ 38; and any value greater than 38 was regarded as a negative test.

### Data analysis

Data analysis was performed using Stata software version 17. The odds ratio (OR) was calculated to assess the role of the detected virus in the case group by comparing the infection status of the case group for a given virus with the infection status of the control group for the same virus. Multivariate logistic regression was used to calculate the adjusted OR (aOR) by adjusting for the presence of other viruses. The association between the mean CT value for a given virus in the case and control group was measured using a Two-sample Wilcoxon rank-sum (Mann–Whitney) test. The attributable fraction among the exposed (AFE) (i.e. the proportion of cases infected with a given virus for whom that virus was deemed responsible for their illness), was calculated as 1 − (1/OR), where OR is the case–control odds ratio for that virus. The population attributable fraction PAF for a given virus (i.e., the proportion of all cases attributable to a given virus) was calculated as (AFE% for a given virus × prevalence of a given virus among cases).

### Ethical review

This study was approved by AHRI and Addis Ababa University ethical review committees. Written and signed informed consent was obtained from all Parent/Guardian of enrolled children.

## Result

### Sociodemographic characteristics

A total of 420 NP/OP swab samples were collected from 210 cases and 210 controls. Table 2 shows a summary of the baseline characteristics of the cases and controls. The median age of children with LRTIs and controls was 15.1 months and 22.8 months, respectively. The proportion of males in the cases and controls was 131(62.4%) and 111(52.9%), respectively. In general, there were no significant baseline characteristics differences between cases and controls population (Table 2).

**Table 2:** Characteristics of cases and controls population

Characteristic	Cases (210) (%)	Controls (210) (%)	*p* Value
Sex (male)	131(62.4)	111(52.9)	0.052
Mean age in month	15.1	22.8	0.109
*Age category (month)*			
0–12	130(62)	97(46.1)	
13–24	49(23)	48(22.7)	0.218
25–36	19(9)	29(13.6)	0.119
37–48	7(3)	18(8.4)	0.056
49–60	5(2)	19(9.1)	0.992
Mean admission weight (KG)	8.5	9.8	0.638
Mean admission height(CM)	68.5	74.8	0.804
Mean mid upper arm circumference(CM)	13.4	12.9	0.194
*Malnutrition status*			
Normal	189(89.9)	185(88.0)	
Moderate acute malnutrition(MAM)	8(3.7)	15(7.2)	
SAM	13(6.4)	10(4.8)	0.578
Breastfeeding practices	195(92.9)	177(84.2)	0.996
HIV/AIDS Positive	2(1.1)	1(0.7)	
*Immunization*			
Immunized all scheduled program	131(62.5)	139(66.1)	
Miss some immunization	8(3.6)	13(6.0)	0.422
Not immunized at all	13(6.3)	10(4.8)	0.122
Immunization on progress (for < 9 months children)	58(27.6)	49(23.2)	0.529
*Exposure to childcare/kindergarten*			
Nursery/pre-KG	3(1.6)	12(5.6)	
KG	8(3.7)	21(9.9)	0.995
No exposure	199(94.7)	178(84.6)	
Child’s mother age	29.2	29.1	0.838
*Maternal education*			
Illiterate	40(19.2)	51(24.1)	
Non-formal education	2(1.1)	1(0.6)	
Primary School	101(48.0)	92(43.8)	0.763
Secondary School	39(18.6)	39(18.5)	0.285
Higher education	27(13.0)	27(13.0)	0.844
*Smokers in household*			
Yes	8(3.7)	4(1.8)	
*Indoor air pollution from solid fuel*			
Yes	116(55.1)	130(62.1)	0.455

### Respiratory viruses associated with LRTIs among children under-five years

**Fig. 1:**
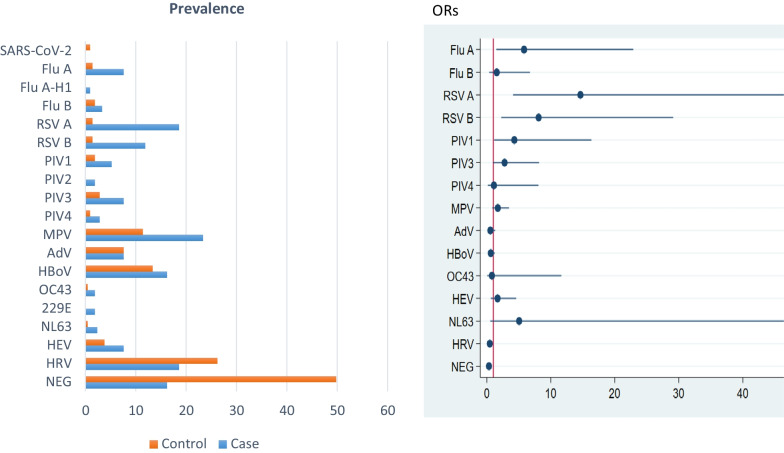
Prevalence of respiratory viruses in nasopharyngeal/oropharyngeal swabs at hospital admission [Left]. ORs with 95% CIs for detection of respiratory viruses in cases compared with controls [Right]. SARS‑CoV‑2 indicates Severe acute respiratory syndrome coronavirus 2; Flu A, Influenza A virus; Flu A-H1, Influenza A-H1; Flu B, Influenza B virus; RSV A, Respiratory syncytial virus A; RSV B, Respiratory syncytial virus B; PIV 1, Parainfluenza virus 1; PIV 2, Parainfluenza virus 2; PIV 3, Parainfluenza virus 3; PIV 4, Parainfluenza virus 4; MPV, Metapneumovirus; AdV, Adenovirus; HBoV, Bocavirus 1/2/3/4; OC43, Coronavirus OC43; 229E, Coronavirus 229E; NL63, Coronavirus NL63; HEV, Enterovirus; HRV, Human rhinovirus; NEG, Negative for all tested viruses

The likelihood of detecting one or more viruses from NP/OP was higher among cases than controls (83.8% vs. 50.3%, *p* = 0.004). The respiratory viruses most frequently detected in both cases and controls were HRV [(n = 39, 18.6%) in cases and (n = 55, 26.2%) in controls)], hMPV [(n = 49, 23.3%) in cases and (n = 24, 11.4%) in controls], and HBoV [(n = 34, 16.2%) in cases and (n = 28, 13.3%) in controls] (Fig. 1, Additional file 1: Table S1). RSV A (n = 39, 18.6%) and RSV B (n = 25, 11.9%) viruses were more commonly detected in the cases. We tested all the samples for SARS-CoV-2 and only two samples from the control group were positive. After adjusting for the presence of other viruses, the multivariate logistic regression showed a significantly higher detection rate for RSV A (OR: 14.6, 95% CI 4.1–52.3), RSV B (OR: 8.1, 95% CI 2.3–29.1), influenza A virus (OR: 5.8, 95% CI 1.5–22.9), and PIV 1 (OR: 4.3, 95% CI 1.1–16.4) among cases when compared with controls.

### Attributable fraction analysis

**Fig. 2:**
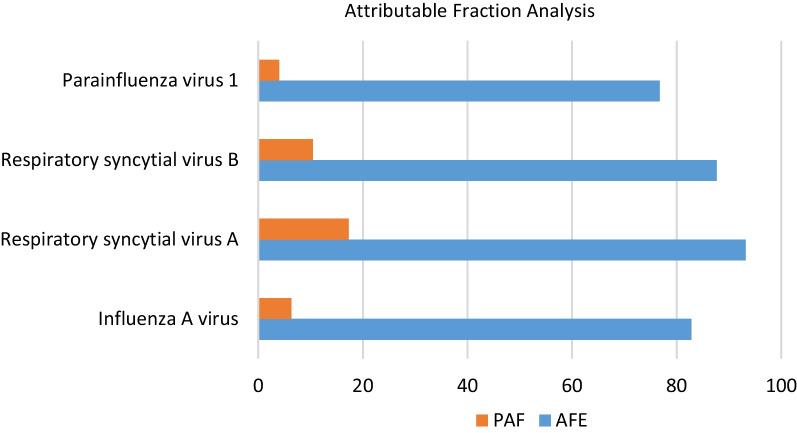
Attributable fraction among the exposed (AFE) and the population attributable fraction (PAF)

We performed attributable fraction among the exposed (AFE) and the population attributable fraction (PAF) analysis for the viruses that showed significant association with LRTI cases. The highest AFE was observed for RSV A with 93.2% (i.e. 93.2% of cases testing positive for RSV A are attributable to RSV A). RSV B had the second-highest AFE with 87.7%, and Influenza A had an AFE of 82.8% (Fig. 2, Additional file 1: Table S2). Population attributable fraction (PAF) was calculated to obtain the fraction of cases attributed to each respiratory virus. The highest PAF was also observed for RSV A with 17.3% (i.e., the proportion of all cases attributable to RSV A was 17.3%). The overall PAFs for RSV B and Influenza A viruses were 10.4% and 6.3% respectively (Fig. 2, Additional file 1: Table S2).

### PCR cycle threshold values in cases and controls

**Fig. 3:**
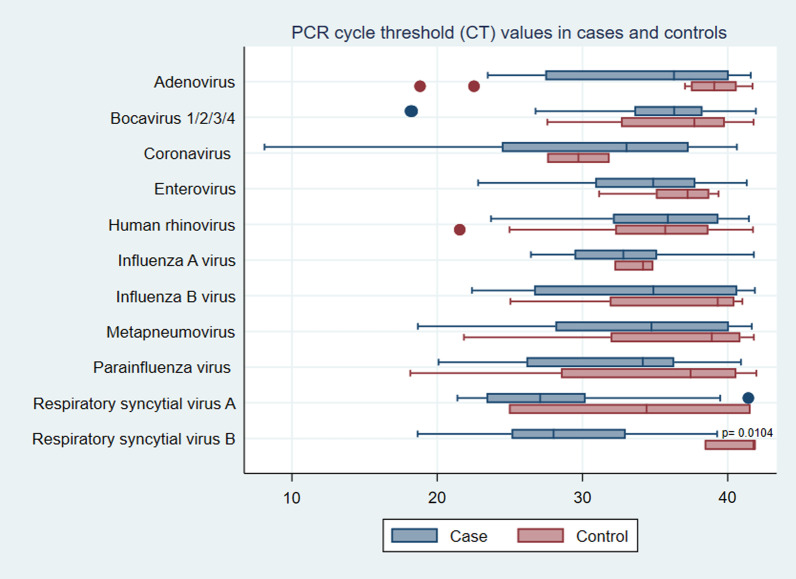
PCR cycle threshold (CT) values in cases and controls. Box plot of CT values for all PCR-positive cases and controls sorted by the virus. *p* Values calculated with Mann–Whitney U test

Cycle threshold (CT) values for each virus were compared between cases and controls as an inverse estimate of viral load (Fig. 3). The mean CT values were lower in all of the viruses detected in the cases with the exception of coronaviruses and human rhinoviruses. However, by p values calculated with a Two-sample Wilcoxon rank-sum (Mann–Whitney) test, only RSV B had significantly lower CT values as compared with controls (*p* = 0.01).

### Age-related prevalence of respiratory viruses

**Fig. 4:**
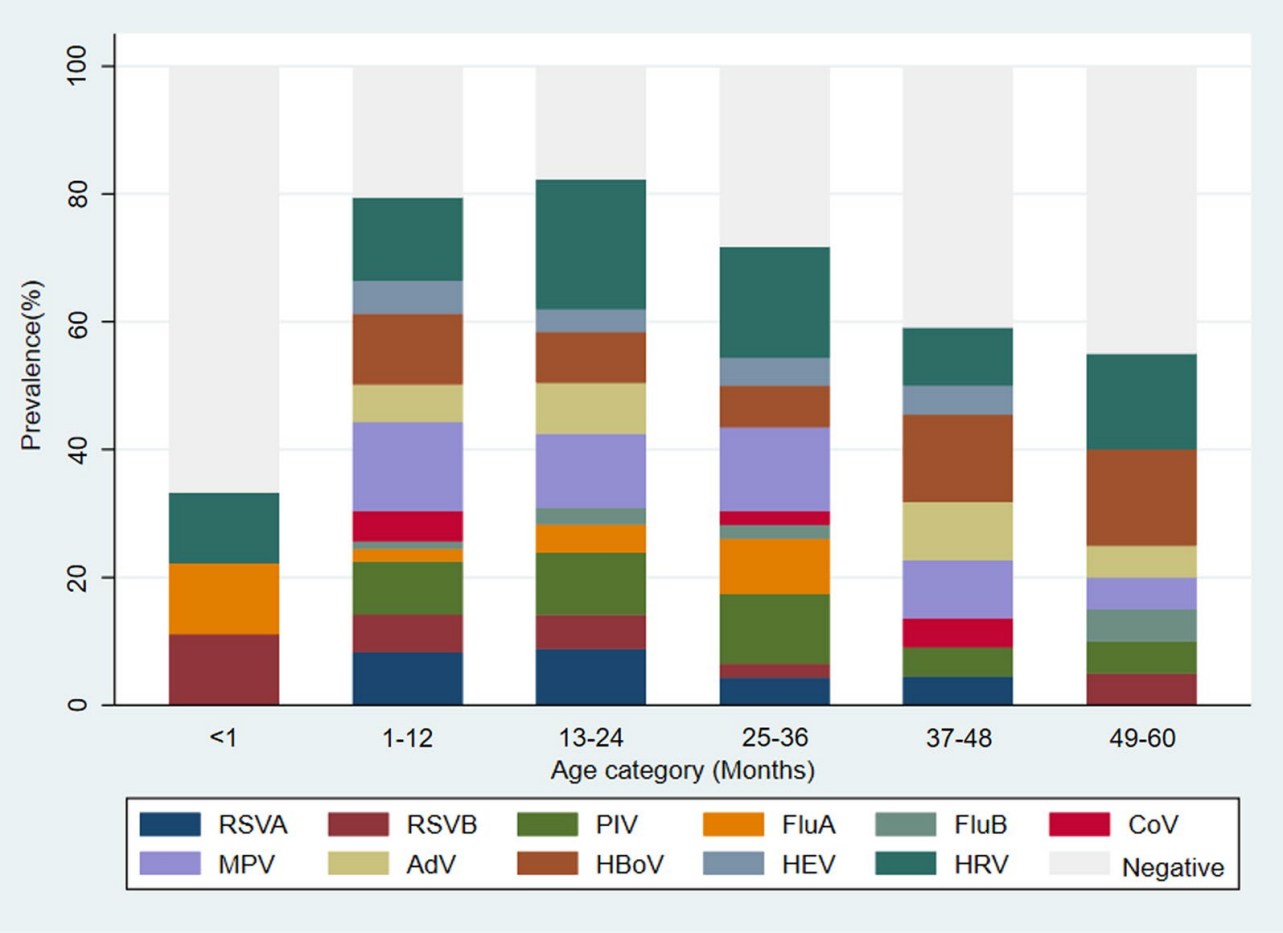
Age-related prevalence of respiratory viruses. Flu A indicates Influenza A virus; Flu B, Influenza B virus; RSV A, Respiratory syncytial virus A; RSV B, Respiratory syncytial virus B; PIV, Parainfluenza virus 1/2/3/4; MPV, Metapneumovirus; AdV, Adenovirus; HBoV, Bocavirus 1/2/3/4; Cov, Coronavirus OC43/Coronavirus 229E/Coronavirus NL63; HEV, Enterovirus; HRV, Human rhinovirus; Negative, Negative for all tested viruses

The distribution of respiratory viruses among different age groups is shown in Fig. 4. The viruses detection rates in the 0–1-month-old group, 1–12-month-old group, 13–24 month-old group, 25–36 month-old group, 37–48-month-old group and 49–60-month-old group were 33.3%(3/9), 79.4%(201/253), 82.3%(93/113), 71.7%(33/46), 59.1%(13/22) and 55.0%(11/20), respectively. However, the differences in the detection rate among the age groups were not statistically significant (*p* > 0.05). In the 0–1-month-old group, the three viruses frequently detected were RSV B, FluA and HRV. RSV A (OR: 3.3, 95% CI 0.97–10.9) and RSV B (OR: 3.4, 95% CI 0.78–14.7) were predominantly obtained from infants and toddlers than preschoolers (children 2–5 years old).

## Discussion

We found that the likelihood of detecting one or more respiratory viruses from the NP/OP of children with LRTIs compared to the control group was higher (83.8% vs. 50.3%). Previously conducted similar studies by Kelly et al., Stephen R. C. et al., Breiman et al., Hammitt et al., and Juliet O et al., also revealed a higher rate of virus detection among cases than controls, with (74.8% vs. 33.8%), (93.1% vs. 79.8%), (72.9% vs. 53.0%), (16.2%vs. 7.4%) and (86.9% vs. 75.4%), respectively [19, 22–26]. Additionally, our results revealed that Influenza A virus, PIV 1, and RSV were associated with LRTIs. Several potential pathogens were identified in children with LRTIs, adding to the evidence that childhood LRTIs and severe disease might often not be due to a single organism [27, 28].

The highest OR were observed for RSV A (OR: 14.6, 95% CI 4.1–52.3) and RSV B (OR: 8.1, 95% CI 2.3–29.1); the corresponding PAF were 17.3% and 10.3%, respectively. The PAF is frequently interpreted as the proportion of disease risk (in our case 17.3% LRTIs cases were attributable to RSV A), that could be eliminated from the population if exposure (RSV A) were eliminated. The PAF has a practical value for those interested in public health prevention policy, particularly when dealing with an exposure that is modifiable through appropriate prevention and treatment strategies [29]. The dominance of RSV was also reported in a number of case–control studies in sub-Saharan Africa among < 5 years of children, in which RSV was most strongly associated with LRTI [8, 19, 22–27, 30–35].

In children, recognition of severe outcomes following influenza virus infection has been comparatively recent [36]. In this study, Influenza A viruses were found predominantly in children with LRTIs compared with controls (OR: 5.8, 95% CI 1.5–22.9). Various studies in sub-Saharan Africa also reported a significant association between Influenza A virus and LRTI [22, 24, 32, 34], while some studies found no significant association [23, 24, 26, 31, 35]. A meta-analysis by Nair et al. [37] estimated that between 28,000 and 111,500 deaths in children aged less than 5 years are attributable to influenza-related causes, the vast majority of which occur in developing countries. Effective vaccines for IFV are available, and the World Health Organization (WHO) strongly recommends pregnant women be vaccinated to protect both the mothers and the infants [38]. Although Ethiopia established influenza sentinel surveillance in 2008 with the aim of knowing the burden and circulating influenza strains in the country, the vaccination program has not yet been introduced [39]. This finding should encourage the acceleration of targeted interventions against Influenza infections in the country.

We also found a significant association of Parainfluenza 1 with LRTIs. A number of studies reported a significant association between Parainfluenza 1 virus and LRTI cases when compared with asymptomatic children or children with mild respiratory infections [22, 25, 33, 34], while others found no difference [8, 23, 24, 31, 35]. We found no association between Parainfluenza 2, 3 and 4 and LRTIs, this finding is consistent with the results of similar case–control studies in sub-Saharan Africa [22, 25, 31–34]. Prevention and treatment strategies targeting RSV, Influenza A virus, and PIV1 may have a beneficiary effect on combating LRTI in children.

We also tested all the NP/OP swab samples for SARS-CoV-2 and found only two positive results from the control group. Large epidemiological studies also reported that children comprise only 1–2% of all SARS-CoV-2 cases [40–42]. Early after the new SARS-CoV-2 was first described in the Hubei province of China, it became clear that most children infected with SARS-CoV-2 were asymptomatic or had mild symptoms [43, 44]. Whether children are also less likely to get infected by SARS-CoV-2 is an ongoing debate. However, more recent studies reported that children are less often infected by SARS-CoV-2 after contact with a SARS-CoV-2-positive individual [44].

Pathogen density in the nasopharynx could provide additional information and may further aid in distinguishing asymptomatic from symptomatic infections [45]. We found that the mean CT values were lower in all of the viruses detected in the cases with the exception of coronaviruses and human rhinoviruses. Generally, children with LRTI had a higher total viral load and harbored more viruses than asymptomatic children or children with mild respiratory infections. A number of studies from sub-Saharan Africa reported higher nasopharyngeal viral density in LRTIs cases compared with non-LRTI controls [14–19]. For some viruses, increased nasopharyngeal viral load has been associated with the clinical severity of LRTIs in children [45].

## Conclusion

These findings underscore the importance of developing prevention strategies for RSV and Influenza A virus in Ethiopia and support the importance of developing an effective vaccine against these viruses. RSV vaccines designed for children are in the pipeline and immediate launch of RSV vaccination soon after approval shall be recommended for children in Ethiopia. The introduction of Influenza A vaccination shall also be recommended. Regarding SARS-CoV-2, only 2 children were positive out of 420 children; therefore, further research should be done before launching a vaccination campaign for < 5 years children against Covid-19 in resource-limited countries like Ethiopia. Further work should be done to inform a reliable CT value cut-off for specific viruses to diagnose LRTIs cases in clinical settings.

## Supplementary Information

**Additional file 1. Table S1. **The prevalence of Respiratory viruses associated with severe acute respiratoryinfections among under-five year’s cases and controls.** Table S2. **Attributable Fraction among Exposed (AFE) and Population Attributable Fraction (PAF).

## Data Availability

The datasets during and/or analyzed during the current study are available from the corresponding author on reasonable request.

## Abbreviations

229E: Coronavirus 229E
AdV: Adenovirus
AFE: Attributable fraction among exposed
AHRI: Armauer Hansen Research Institute
Ct: Cycle threshold
Flu A: Influenza A virus
Flu A-H1: Influenza A-H1
Flu A-H1pdm09: Influenza A-H1pdm09
Flu A-H3: Influenza A-H3
Flu B: Influenza B virus
HBoV: Bocavirus 1/2/3/4
HEV: Enterovirus
HRV: Human rhinovirus
LRTI: Lower respiratory tract infection
MPV: Metapneumovirus
NL63: Coronavirus NL63
NP/OP: Naso/Oropharyngeal
OC43: Coronavirus OC43
OR: Odds ratio
PAF: Population attributable fraction
PIV 1: Parainfluenza virus 1
PIV 2: Parainfluenza virus 2
PIV 3: Parainfluenza virus 3
PIV 4: Parainfluenza virus 4
RSV A: Respiratory syncytial virus A
RSV B: Respiratory syncytial virus B
SARS-CoV-2: Severe acute respiratory syndrome coronavirus 2
