# Supplementary material for: Viral etiologies of lower respiratory tract infections in children < 5 years of age in Addis Ababa, Ethiopia: a prospective case–control study
Source: Virol J. 2023 Jul 23;20:163. doi: 10.1186/s12985-023-02131-x (PMC10363322; doi:10.1186/s12985-023-02131-x)
Supplement: Supplementary file 1 — Additional file 1. Table S1. The prevalence of Respiratory viruses associated with severe acute respiratoryinfections among under-five year’s cases and controls. Table S2. Attributable Fraction among Exposed (AFE) and Population Attributable Fraction (PAF). [file 12985_2023_2131_MOESM1_ESM.docx]

| **Virus** | **Case%** | **Control%** | **Case** | **Control** |
| --- | --- | --- | --- | --- |
| Negative for all tested virus | 16.19047619 | 50 | 34 | 105 |
| Human rhinovirus | 18.57142857 | 26.19047619 | 39 | 55 |
| Enterovirus | 7.619047619 | 3.80952381 | 16 | 8 |
| Coronavirus NL63 | 2.380952381 | 0.476190476 | 5 | 1 |
| Coronavirus 229E | 1.904761905 | 0 | 4 | 0 |
| Coronavirus OC43 (OC43) | 1.904761905 | 0.476190476 | 4 | 1 |
| Bocavirus 1/2/3/4 | 16.19047619 | 13.33333333 | 34 | 28 |
| Adenovirus | 7.619047619 | 7.619047619 | 16 | 16 |
| Metapneumovirus | 23.33333333 | 11.42857143 | 49 | 24 |
| Parainfluenza virus 4 | 2.857142857 | 0.952380952 | 6 | 2 |
| Parainfluenza virus 3 | 7.619047619 | 2.857142857 | 16 | 6 |
| Parainfluenza virus 2 | 1.904761905 | 0 | 4 | 0 |
| Parainfluenza virus 1 | 5.238095238 | 1.904761905 | 11 | 4 |
| Respiratory syncytial virus B | 11.9047619 | 1.428571429 | 25 | 3 |
| Respiratory syncytial virus A | 18.57142857 | 1.428571429 | 39 | 3 |
| Influenza B virus | 3.333333333 | 1.904761905 | 7 | 4 |
| Influenza A-H1 | 0.952380952 | 0 | 2 | 0 |
| Influenza A virus | 7.619047619 | 1.428571429 | 16 | 3 |
| SARS-CoV-2 | 0 | 0.952380952 | 0 | 2 |

Table 1. The prevalence of Respiratory viruses associated with severe acute respiratory infections among under-five year’s cases and controls

Table 2. Attributable Fraction among Exposed (AFE) and Population Attributable Fraction (PAF)

| Virus | AFE | PAF |
| --- | --- | --- |
| Influenza A virus | 82.81828 | 6.309964 |
| Respiratory syncytial virus A | 93.16695 | 17.30243 |
| Respiratory syncytial virus B | 87.65318 | 10.4349 |
| Parainfluenza virus 1 | 76.74677 | 4.020069 |
